# Health Tract, No. 119

**Published:** 1862-11

**Authors:** 


### HEALTH TRACT, No. 119.

### THAT BEST DAY.

"WHAT are you going to do now?" said a gentleman to his friend on Broadway, who had recently failed in business. "I believe I will go home and get acquainted with my family," was the reply.

There is a man in this city known on both continents. He assured a friend one day that for nearly seven years he had not seen any of his family out of bed, except on Sundays. He ate breakfast at sunrise, hurried down-town, took dinner at Delmonico's, and returned late at night to find all in bed. So wholly was he engrossed in business, so absorbed in money-making, that all family ties, all its affections, all its loves, were of secondary importance. *His* "chief end" was to get rich! He succeeded; but at a cost of heart-warmth, of the luscious loves of infancy and childhood, which made it a dear bargain. But what became of his sworn duty to his wife all this time — the great duty of sympathy in the burdens of housekeeping and child-training, duties which no man can permit to remain in abeyance without committing a crime against his family, against society, and against the great Father of all, who has intrusted the proper training of children to parental care? What was the result of these great derelictions? This man failed; lost every dollar of his fortune; strove again for wealth, succeeded, and again failed. For the third time he failed, and at this writing is not worth a dime.

Both these cases show that the pursuit of wealth in large cities becomes an infatuation, a frenzy, which bears down the victim of unhallowed greed so resistlessly, that he becomes unconscious of the highest obligations of humanity; his moral sense grows so obtunded that he sees nothing, feels nothing, hears nothing but what pertains to the getting of money. Is it right? will reason approve of it? will humanity approve of it? will an outraged conscience approve of it in the terrible hour of the last conflict with death? This ignoring of all obligations, human and divine, in the crazed pursuit of riches, does not largely obtain, except in the great cities of the world, where human ambitions are stimulated by rivalries to the intensest pitch. Still the onward rush for wealth is like the dashing of an infuriated steed down a steep declivity—every moment and every step but increase the momentum; and the human tide would be numbered by millions in every grade of life, in the country as well as in the town, did not infinite benevolence "put down the breaks" at short intervals by the blessed institution of the Sabbath day, which a poor laborer, with beautiful truthfulness, once called "that best day," because it was all his own; because on other days he was expected to work for his employer from early dawn until the darkness, when he was too tired himself and his children were too sleepy for the interchange of affectionate caresses. But when the Sabbath came it was a day of resting, and in contemplation of the privilege of being with his family through the whole of it, either around the fireside, at the family table, or at the village-church, he felt it was "that best day" of all the seven. It is physiologically the "best day," because it is a necessary rest for both brain and body; necessary for man, necessary for beast, hence Divinity has ordered, "In it thou shalt not do any work; thou, nor thy son, nor thy daughter, nor thy man-servant, nor thy maid-servant, nor thy cattle," and the man who, in the light of the Bible, persistently and systematically violates this command, lovingly intended for his best good, physical and moral, may reasonably expect the Almighty's signal punishment, either in the failure of his earthly ambitions, the premature failure of the vital powers, or that greater failure still, the blasting of the mind.

